# ASH1L contributes to oocyte apoptosis by regulating DNA damage

**DOI:** 10.1152/ajpcell.00196.2022

**Published:** 2022-09-12

**Authors:** Tuo Zhang, Tianhe Ren, Huan Lin, Yuntong Tong, Jixian Zhang, Jie Nie, Yingjie Zhu, Yiting Wang, Bangming Jin, Chunlin Zhang, Tengxiang Chen, Meina He

**Affiliations:** ^1^Transformation Engineering Research Center of Chronic Disease Diagnosis and Treatment, Department of Physiology, College of Basic Medicine, Guizhou Medical University, Guiyang, Guizhou Province, China; ^2^College of Basic Medicine, Guizhou Medical University, Guiyang, Guizhou Province, China; ^3^Affiliated Hospital of Guizhou Medical University, Guiyang, Guizhou Province, China; ^4^Guizhou Institute of Precision Medicine, Affiliated Hospital of Guizhou Medical University, Guiyang, Guizhou Province, China

**Keywords:** apoptosis, ASH1L, DNA damage checkpoint, oocyte, primordial follicle

## Abstract

In female mammals, the size of the initially established primordial follicle pool within the ovaries determines the reproductive life span. Interestingly, the establishment of the primordial follicle pool is accompanied by a remarkable programmed oocyte loss for unclear reasons. Here, we identify a new role of ASH1-like histone lysine methyltransferase (ASH1L) in controlling the apoptosis of oocytes during meiotic prophase I in mice. Our results showed that overexpression of *Ash1l* led to a dramatic loss of fetal oocytes via apoptosis, which subsequently resulted in a reduced capacity of the primordial follicle pool. Overexpression of *Ash1l* also led to a deficiency in DNA double-strand break repair associated with premature upregulation of p63 and phosphorylated checkpoint kinase 2 (p-CHK2), the major genome guardian of the female germline, following *Ash1l* overexpression in fetal ovaries. In summary, ASH1L is one of the indispensable epigenetic molecules that acts as a guardian of the genome. It protects oocyte genome integrity and removes oocytes with serious DNA damage by regulating the expression of p63 and p-CHK2 during meiotic prophase I in mice. Our study provides a perspective on the physiological regulatory role of DNA damage checkpoint signaling in fetal oocyte guardianship and female fertility.

## Graphical abstract

**Figure:**
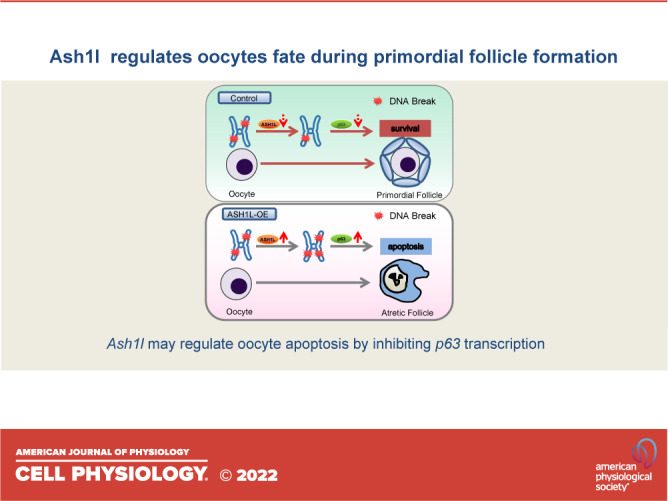

## INTRODUCTION

The primordial germ cell migrates to the genital ridge at 10.5 days postcoitus (dpc) in mouse, and then it starts to undergo rapid mitosis and forms a germ cell cyst structure (1, 2). At ∼13.5 dpc, some of the germ cells, which are also called oocytes, start to go into meiosis and under four stages of meiotic prophase I including the leptotene, zygotene, pachytene, and diplotene stages in females. At 17.5 dpc, oocytes become arrested in the dictyate stage and the pregranulosa cells begin to encase a single oocyte, forming a primordial follicle structure. The primordial follicle pool is established at ∼3–5 days postpartum (dpp) in mice (3, 4). Two-thirds of the oocytes are abolished during primordial follicle formation (5–7).

Apoptosis plays an important role in oocyte loss. Current findings show that apoptosis regulates oocyte fate and follicular pool size and most of the regulation occurs at the initial stage of the first division of meiosis and the germ cell cysts break down (8–10). The meiosis process is abnormally arrested or DNA damage cannot be repaired and can lead to oocyte apoptosis. After some DNA damage, repair gene mutations, such as ATM serine/threonine kinase (ATM) and RAD51 recombinase (RAD51), can cause oocyte apoptosis (11, 12). To investigate the role of apoptosis in oocyte death, some genes that regulate apoptosis were knocked down. Studies have shown that BCL2 Binding Component 3 (*BBC3*) deletion increased the number of oocytes significantly in the embryonic period of mice (13), so it is very important to explore the role of apoptosis in primordial follicle formation.

In female mammals, the ovarian follicles are the basic and nonrenewable reproductive unit that determines the reproductive life span. The formation of primordial follicles in fetal ovaries and newborn ovaries is under precise control (14). Histone methylation plays important roles during the follicle development and oocyte maturation process (15, 16). Altered histone methylation changes the developmental fate of follicles. Inadequate follicles in the initially established pool are related to primary premature ovarian insufficiency (POI) (17, 18). Therefore, an in-depth study of histone methylases regulating fetal oocyte maintenance and attrition will help us better understand the pathogenic mechanism of POI and female infertility in mammals, but the underlying mechanism that regulates oocyte sustainment and elimination is not fully understood.

ASH1L is a H3-methyltransferase that belongs to the Trithorax group family (19, 20). It has been reported to methylate H3K4 and H3K36 (21, 22). It was reported that *Ash1l* mutant mice exhibit partially postnatal lethality and other mutant mice that survive have growth insufficiency and infertility caused by defects in both epididymis and uterine development in male and female mice (23). Studies demonstrated that knockdown of *Ash1l* in bovine cumulus cells induced a decrease in the levels of H3K36me1/2/3, inhibited proliferation, and upregulated the expression of apoptosis (24). However, the role of ASH1L in meiotic prophase I and early folliculogenesis during the fetal stage remains unclear in mammals. Whether ASH1L plays a crucial role in fetal oocyte development requires comprehensive exploration.

In this study, we clarified the function of ASH1L in sustaining fetal oocyte survival and the formation of primordial follicles in the perinatal mouse ovary. Overexpression of *Ash1l* leads to premature apoptosis of oocytes with persistent unrepaired double-strand breaks (DSBs) in oocytes. The underlying mechanism revealed that overexpression of *Ash1l* induced premature p63 expression and initiated apoptosis during meiotic prophase I.

## MATERIALS AND METHODS

### Animals

All male and female CD1 mice were purchased from Beijing Vital River Laboratory Animal Technology Co., Ltd. (Beijing, China). Female mice were mated with males overnight and checked for a vaginal plug the following morning. The presence of a vaginal plug was considered 0.5 day postcoitus (dpc). The day after partum was considered to be 1 day postpartum (dpp). All animal experiments conformed to the guidelines and regulatory standards of the Institutional Animal Care and Use Committee of Guizhou Medical University. All mice were anesthetized by intraperitoneal injection of 1% pentobarbital sodium (50 mg/kg).

### Ovary Isolation and Culture

16.5 dpc female mouse ovaries were separated by microdissection from the mesonephros or ovarian capsule in pre-chilled PBS (10 mM, pH 7.4) under a stereomicroscope (ZSA302; COIC, China) at the same time. The isolated ovaries were cultured in six-well culture dishes (NEST Biotechnology, China) with basic DMEM-F-12 medium (Gibco, Life Technologies, United States) and penicillin-streptomycin at 37°C in a 5% CO_2_, 95% air atmosphere with saturated humidity. Ovaries were cultured in either medium with dimethyl sulfoxide (DMSO) or medium supplemented with inhibitor. The culture medium was exchanged every other day. The final concentration of Z-VAD-fmk was 50 μM.

### Plasmid Construction

Gene coding area of *Ash1l* was cloned into pCMV-Blank (D2602; Beyotime, China). Golden star t6 super PCR mix was purchased from Beijing Tsingke Co., Ltd. All of the constructs were verified by sequencing. Primers are listed in Supplemental Table S1.

### Gene Overexpression

To assure that overexpression vectors would be transfected into the inner cells of ovaries, 0.5 μL of the overexpression vectors was injected into isolated 16.5 dpc female mouse ovaries with glass pipettes and a stereomicroscope. After the ovaries were full of liquid, electrotransfection (ECM2001; BTX, California) was performed by applying three 5-ms-long quasi-square pulses at a pulse-field strength of up to 30 V/cm. Ovaries were cultured for 72 h and collected at the same time to test the transfection efficiency of protein levels or for 6 days for histological examination and oocyte counting. Primers used are listed in Supplemental Table S1.

### Histological Sections and Oocyte Counts

The ovaries of control and treatment groups were collected at the same time; ovaries were fixed in cold 4% paraformaldehyde overnight, embedded in paraffin, and serially sectioned at 5 μm. The sections were stained with hematoxylin, and the number of oocytes was counted in every fifth section. To estimate the total numbers of oocytes in each ovary, the sum was multiplied by 5. Sections were examined and photographed with a Nikon A1 laser scanning confocal microscope. Immunohistochemistry (IHC) photos were taken in a light environment at 22°C, with a ×20 objective lens. All the images were obtained with NIS-Elements Viewer 4.50 software, combined with Photoshop CS6. Intensity and/or contrast was not changed in all the pictures of control and treatment groups.

### Immunofluorescence

The ovaries of control and treatment groups were collected at the same time; ovaries were fixed in 4% paraformaldehyde overnight, embedded in paraffin, and sectioned at 5 μm. After dewaxing, rehydration, and high-temperature (92°C) antigen retrieval with 0.01% sodium citrate buffer (pH 6.0), the sections were blocked with 10% normal serum for 60 min at room temperature and immunostained with primary antibodies overnight at 4°C. Source and dilutions of primary antibodies are presented in Supplemental Table S2. Validation of the primary antibody specificity is shown in Supplemental Material. Subsequently, after thorough rinsing with PBS, the slides were then incubated with Alexa Fluor 488- or 555-conjugated secondary antibodies (1:100; Beyotime Biotechnology, China) and DAPI (1:1,000, C1011; Beyotime Biotechnology, China) at 37°C for 1 h. Slides were then rinsed in PBS and sealed in antifade fluorescence mounting medium (Applygen, China) with coverslips.

Sections were examined and photographed with a Nikon Eclipse 80i digital fluorescence microscope or a Nikon A1 laser scanning confocal microscope. Among all the immunofluorescence photos, Fig. 6*E* was taken with a Nikon Eclipse 80i digital fluorescence microscope in a dark environment at 22°C, with a ×100 objective lens. Other immunofluorescence photos were taken with a Nikon A1 laser scanning confocal microscope in a dark environment at 22°C, with a ×20 objective lens. All the images were obtained by NIS-Elements Viewer 4.50 software and analyzed by Photoshop CS6. Intensity and/or contrast was not changed in all the pictures of control and treatment groups.

### Western Blotting

The total protein of the control and treatment groups was extracted at the same time and quantified by bicinchoninic acid (BCA) assay (P0012S; Beyotime Biotechnology, China). Ten milliliters of 10% SDS-PAGE gel was made with 3.3 mL of 30% Acr/Bis (29:1), 2.5 mL of 1.5 M Tris·HCl (pH 8.8), 100 μL of 10% SDS, 100 μL of 10% PAGE gel coagulant, 10 μL of PAGE gel accelerator, and 4.0 mL of ddH_2_O (SDS-PAGE Gel Kit, P1200; Solarbio, China). Electrophoresis and transfer solutions were made as follows: electrophoresis solution: 7.2 g glycine, 1.51 g Tris, and 0.5 g sodium dodecyl sulfate; add 500 mL of deionized water and stir evenly; transfer solution: 14.4 g glycine, 3.02 g Tris base, 800 mL deionized water; add 200 mL of anhydrous methanol solution before use and mix well.

Ten to twenty micrograms of total protein per well was separated on a 10% SDS-PAGE gel and then transferred onto polyvinylidene fluoride membranes (IPVH00010; Millipore, United States). SDS-PAGE and immunoblots were performed following standard procedures with a Mini-PROTEAN Tetra Cell System (Bio-Rad). Source and dilutions of primary antibodies are presented in Supplemental Table S2. The secondary antibodies were horseradishase-conjugated goat anti-mouse IgG (H + L) (ZB-2305; Zhongshan Golden Bridge, China) and horseradishase-conjugated goat anti-rabbit IgG (H + L) (ZB-5301; Zhongshan Golden Bridge, China), diluted at 1:10,000. Detection with chemiluminescence was performed with the Tanon 5200 multichemiluminescence imaging system (Biotaton, China), and quantification was done by dividing all values by the mean of the controls after grayscale scanning.

### Real-Time qPCR

Total RNA was isolated from mouse ovaries with TRIzol (Invitrogen, Life Technologies, United States). One microgram of total RNA was used to synthesize cDNA according to the manufacturer’s instructions (RevertAid First Strand cDNA Synthesis Kit, Kl622; Thermo Scientific, United States). qRT-PCR was performed with TB Green Premix Ex Taq II (RR820A; Takara, United States) with the Bio-Rad Real-Time PCR system. Primers are listed in Supplemental Table S1.

### Chromosome Spreads

SYCP3 antibody was used to identify the chromosomal axial elements at meiosis prophase I. The stages of meiotic prophase I were evaluated based on the appearance of axial elements according to previous studies (25). In total, 300 oocytes from three ovaries were counted on each slide and repeated for three animals. The primary antibodies are presented in Supplemental Table S2.

### Statistical Analysis

Statistical analysis was performed with SigmaPlot 13.0. All experiments were repeated at least three times, and the values are presented as means ± SD. The data were analyzed by *t* test and two-way ANOVA and considered statistically significant at *P* < 0.05.

## RESULTS

### ASH1L Expression Was Increased before and after Birth in Mice

**Figure 1.**
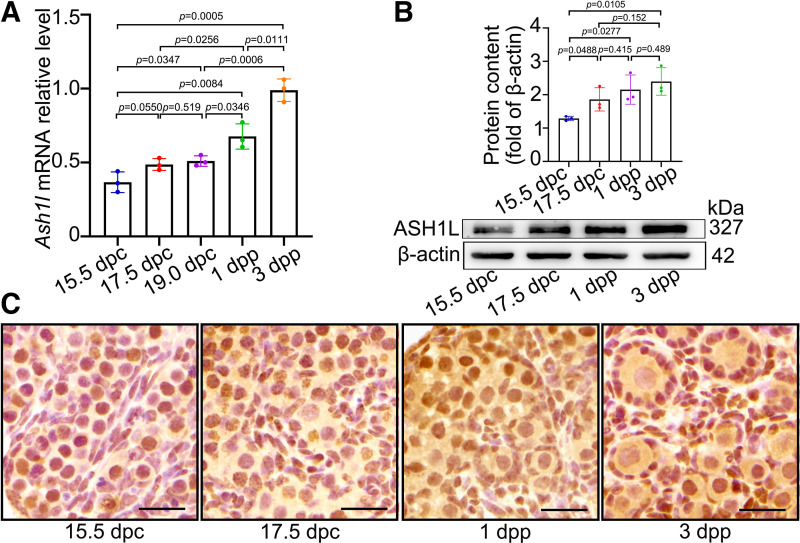
ASH1-like histone lysine methyltransferase (ASH1L) expression pattern in mouse ovaries from 15.5 days postcoitus (dpc) to 3 days postpartum (dpp). *A*: the total mRNA level of *Ash1l* in female mouse ovaries from 15.5 dpc to 3 dpp. *n* = 3. *B*: the total protein level of ASH1L in female mouse ovaries from 15.5 dpc to 3 dpp; every group was 10 ovaries, repeated at least 3 times. *C*: ASH1L was mainly localized to the nucleus of the oocytes and somatic cells. The data were analyzed by *t* test and 2-way ANOVA. Data are shown as means ± SD and considered statistically significant at *P* < 0.05. Scale bars, 25 μm.

To address the physiological function of ASH1L during oocyte loss and primordial follicle formation, we first analyzed the expression pattern and cellular localization of ASH1L during primordial follicle formation. RT-qPCR and Western blotting revealed that the expression level of ASH1L in the ovaries was increased in a time-specific manner from 15.5 dpc to 3 dpp (Fig. 1, *A* and *B*). Immunohistochemistry results showed that ASH1L was located in the nucleus of both somatic cells and oocytes in fetal and neonatal mouse ovaries (Fig. 1*C*). This dynamic expression pattern of ASH1L motivated us to study its potential roles in massive oocyte loss during primordial follicle formation.

### ASH1L Protein Level Determines the Survival of Oocytes around the Time of Birth in Mice

**Figure 2.**
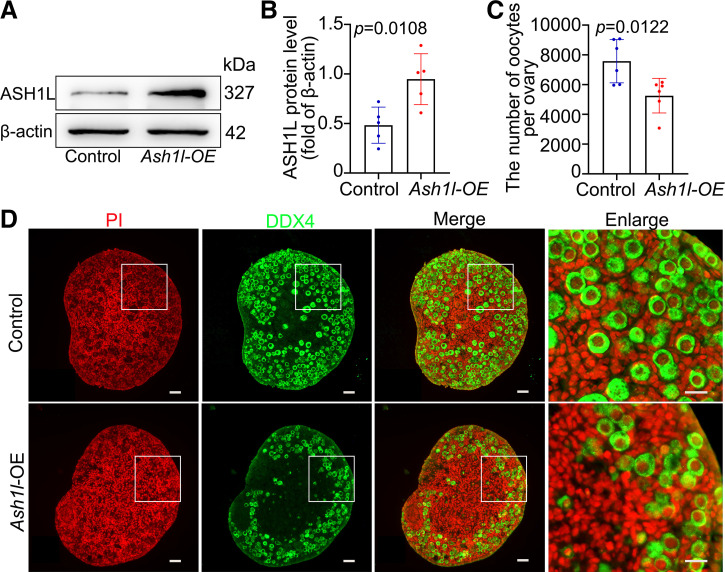
Overexpression of *Ash1l* resulted in massive loss of oocytes. *A* and *B*: *Ash1l* overexpression (OE) efficiency detected by Western blotting. 16.5 days postcoitus (dpc) ovaries were cultured for 3 days in vitro. Every group was 10 ovaries, repeated 5 times. *C* and *D*: overexpression of *Ash1l* led to dramatic oocyte loss in mouse ovaries. 16.5 dpc ovaries were cultured for 6 days in vitro. Oocytes were stained with DDX4 (green). The nucleus was stained with propidium iodide (PI, red). Ovaries in each group were counted; statistical analysis showed that the total number of oocytes was decreased after *Ash1l* was overexpressed. *n* = 6. The data were analyzed by *t* test and 2-way ANOVA. Data are shown as means ± SD and considered statistically significant at *P* < 0.05. Scale bars, 50 μm.

In our previous paper (7), we found that knockdown of *Ash1l* resulted in the survival of more oocytes, but the underlying mechanism is unclear. To further study the function and mechanism of ASH1L, 16.5 dpc ovaries were transfected with the *Ash1l* overexpression (*Ash1l*-OE) plasmid and a scrambled plasmid sequence as the control. The expression efficiency of the transfected plasmids was confirmed by Western blotting after culture for 3 days (Fig. 2, *A* and *B*). *Ash1l*-OE ovaries contained significantly fewer primordial follicle than the control ovaries after 6 days of culture (Fig. 2, *C* and *D*).

### Apoptosis Is Involved in Massive Oocyte Death after *Ash1l* Is Overexpressed

**Figure 3.**
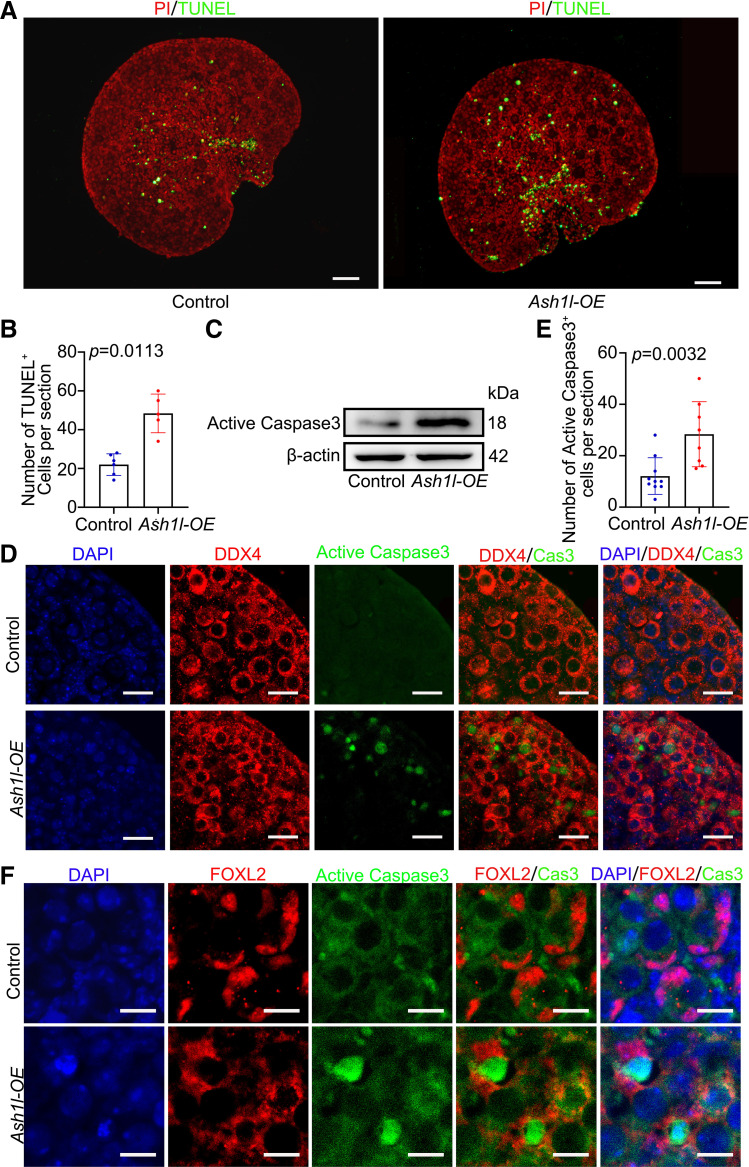
Overexpression (OE) of *Ash1l* resulted in oocyte apoptosis. *A*: positive TUNEL signals (green) corresponded to apoptotic cells; the nucleus was stained with propidium iodide (PI, red). Scale bars, 50 μm. *B*: statistical analysis showed that the number of TUNEL^+^ cells per section increased significantly compared with the control. *n* = 5. *C*: the protein level of active Caspase3 detected by Western blotting. 16.5 days postcoitus (dpc) ovaries were cultured for 3 days in vitro. Every group was 10 ovaries, repeated at least 3 times. *D*: active Caspase3 signals (green) corresponded to apoptotic cells. Oocytes were stained with DDX4 (red). The nucleus was stained with DAPI (blue). Scale bars, 30 μm. *E*: statistical analysis showed that the number of apoptotic oocytes per section increased significantly compared with control. *n* = 8. *F*: active Caspase3 signals (green) corresponded to apoptotic cells. Granulosa cells were stained with FOXL2 (red). The nucleus was stained with DAPI (blue). The data were analyzed by *t* test and 2-way ANOVA. Data are shown as means ± SD and considered statistically significant at *P* < 0.05. Scale bars, 30 μm.

**Figure 4.**
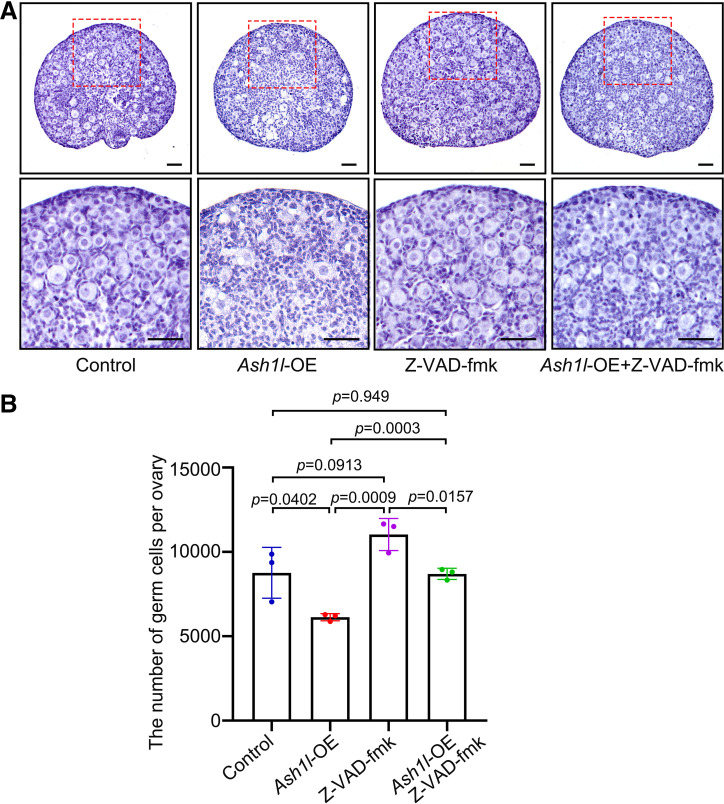
Inhibition of apoptosis could rescue oocyte loss caused by overexpression (OE) of *Ash1l. A*: the rescue of oocytes number by Z-VAD-fmk in *Ash1l*-overexpressing ovaries. Scale bars, 50 μm. *B*: statistical analysis showed that the number of oocytes in Z-VAD-fmk- and *Ash1l* overexpression-treated ovary was rescued significantly compared with the *Ash1l* overexpression ovary. *n* = 3. Data are shown as means ± SD and considered statistically significant at *P* < 0.05.

To clarify whether apoptosis was involved in the oocyte loss, we examined the protein level in the apoptosis pathway in the following assays. We applied the TUNEL assay on the *Ash1l-*OE ovaries. The results showed that the number of TUNEL-positive cells was increased after *Ash1l* was overexpressed (Fig. 3, *A* and *B*). The level of active Caspase3 was examined. Western blotting results showed that the expression level of active Caspase3 was obviously higher in *Ash1l*-OE ovaries than in the control (Fig. 3*C*). Immunofluorescence results showed that active Caspase3 was specifically localized in the nucleus of oocytes, indicating that overexpression of *Ash1l* caused oocyte apoptosis (Fig. 3, *D*–*F*). To provide more evidence demonstrating that apoptosis is pivotal for oocyte loss perinatally, we used a pan-caspase inhibitor, Z-VAD-fmk. We performed a combined assay including control, Z-VAD-fmk, *Ash1l*-OE, and Z-VAD-fmk plus *Ash1l*-OE. Z-VAD-fmk significantly restored the effect of *Ash1l-*OE on oocyte number (Fig. 4). In conclusion, apoptosis leads to oocyte death after *Ash1l* overexpression.

### Overexpression of *Ash1l* Resulted in Abnormal Expression of Genes Related to DNA Damage and Repair

**Figure 5.**
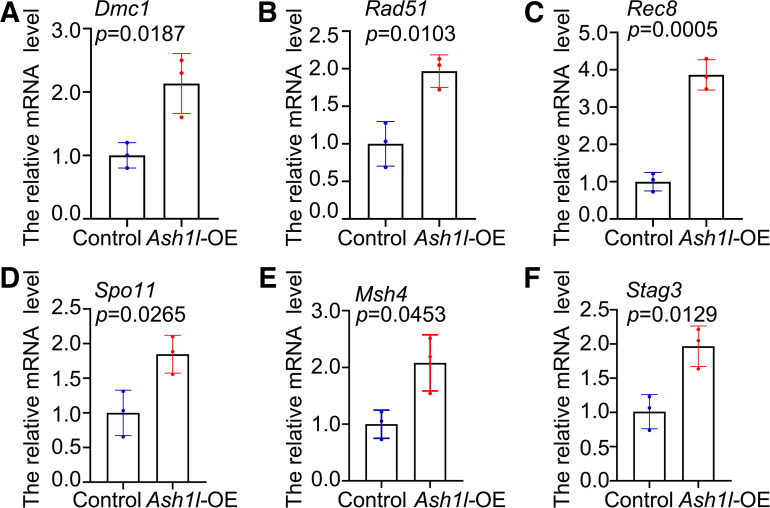
Elevated ASH1-like histone lysine methyltransferase (ASH1L) promotes meiosis-specific gene transcription. *A–F*: the overexpression (OE) of *Ash1l* significantly increased the transcription level of *Dmc1* (*A*), *Rad51* (*B*), *Rec8* (*C*), *Spo11* (*D*), *Msh4* (*E*), and *Stag4* (*F*). Ovaries in the *Ash1l*-OE group exhibited fewer oocytes (arrows) than exhibited by the control. 16.5 days postcoitus (dpc) ovaries were cultured for 3 days in vitro. *n* = 3. The data were analyzed by *t* test and 2-way ANOVA. Data are shown as means ± SD and considered statistically significant at *P* < 0.05.

During primordial follicle formation, DNA damage would cause oocyte apoptosis. To explore the cause of oocyte apoptosis, we examined several DNA damage and repair-related genes. 16.5 dpc ovaries were transfected with the *Ash1l*-OE plasmid and a scrambled sequence plasmid as the control. Ovaries were cultured for 3 days. RT-qPCR results showed that *Dmc1*, *Rad51*, *Rec8*, *Spo11*, *Msh4*, and *Stag3* were significantly changed (Fig. 5, *A*–*F*, respectively). These results indicated that overexpression of *Ash1l* may cause DNA damage.

### Elevated ASH1L Protein Level Promotes Oocyte DNA Damage

**Figure 6.**
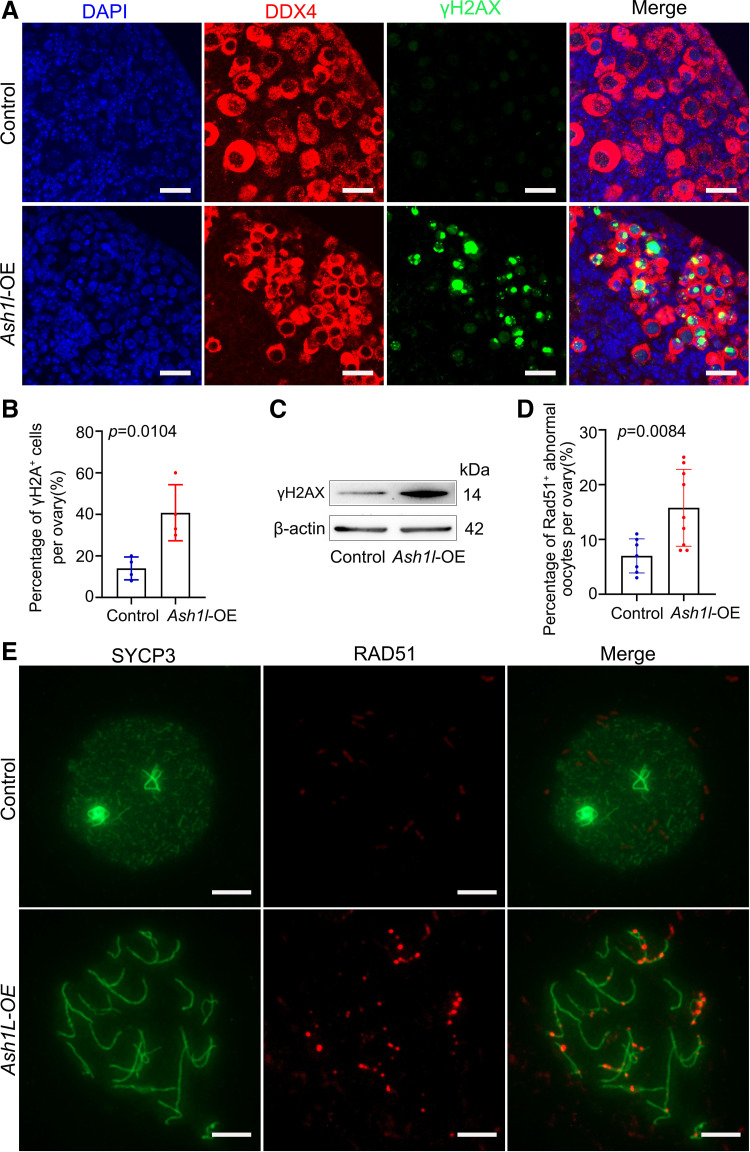
Overexpression (OE) of *Ash1l* resulted in serious oocyte double-strand breaks (DSBs). *A*: the expression level of γH2AX (green) was examined by immunofluorescence after *Ash1l* overexpression. Oocytes were stained by DDX4 (red). The nucleus was stained by DAPI (blue). Scale bars, 25 μm. *B*: statistical analysis showed that the percentage of oocytes with unrepaired DSBs in the diplotene stage on chromosomes per slide increased significantly after *Ash1l* overexpression. *n* = 7. *C*: the protein level of γH2AX was examined by Western blotting after *Ash1l* overexpression. Every group was 10 ovaries, repeated at least 3 times. *D* and *E*: overexpression of *Ash1l* resulted in meiotic DSB repair deficiency. *E*: representative images of the meiotic spread of the diplotene-stage oocytes with normal or ectopic RAD51 foci. Oocyte chromosomes were costained with RAD51 (green) and SYCP3 (red). Scale bars, 10 μm. *D*: statistical analysis showed that the percentage of oocytes with ectopic RAD51 foci on chromosomes in the diplotene stage per slide increased significantly after *Ash1l* overexpression. *n* = 5. The data were analyzed by *t* test and 2-way ANOVA. Data are shown as means ± SD and considered statistically significant at *P* < 0.05.

To detect the oocyte DNA damage, we examined it by histone H2AX phosphorylated at Ser139 (referred to as γH2AX), which marked DSBs on chromatin. 16.5 dpc ovaries were transfected with the *Ash1l*-OE plasmid and a scrambled plasmid sequence as the control. Immunofluorescence results showed that more γH2AX puncta were located in oocyte nuclei compared with the control (Fig. 6, *A* and *B*, Supplemental Fig. S1). Western blotting results showed that γH2AX was elevated in *Ash1l*-OE ovaries (Fig. 6*C*). Moreover, immunofluorescence costaining for RAD51 recombinase (a RecA homolog; a key factor in homologous recombination repair) and SYCP3 demonstrated that partial fetal oocytes showed ectopic RAD51 foci on the pachytene chromosome following overexpression of *Ash1l*, which implied incomplete DSB repair in the oocytes in *Ash1l*-OE ovaries (Fig. 6, *D* and *E*). In summary, overexpression of *Ash1l* resulted in abnormal meiotic DSB repair and meiotic progression errors.

### The Upregulation of p63 and p-CHK2 Causes Oocyte Apoptosis after *Ash1l* Is Overexpressed

**Figure 7.**
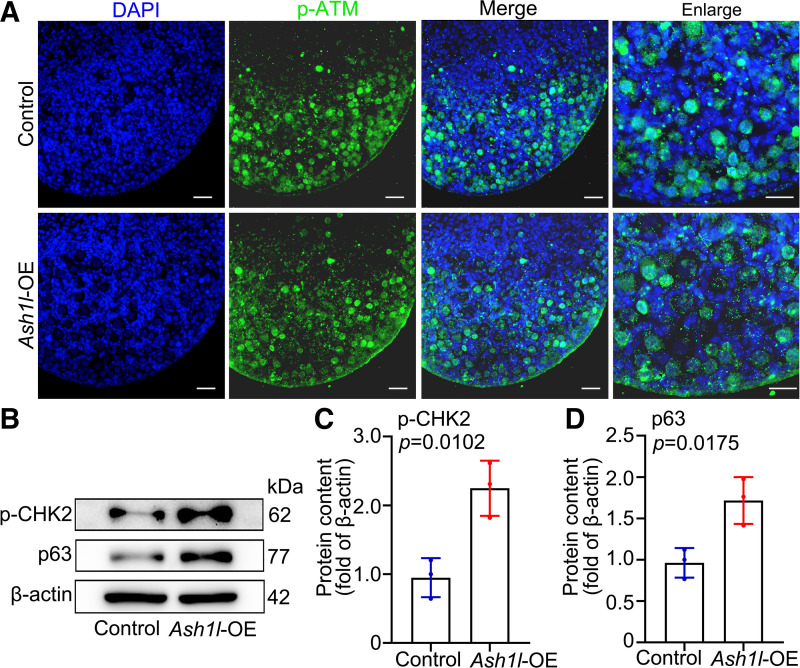
Overexpression (OE) of *Ash1l* upregulation of p63 level resulted in massive oocyte loss in mice. *A*: the expression level of phosphorylated ataxia telangiectasia mutated kinase (p-ATM, green) was examined by immunofluorescence after *Ash1l* overexpression. The nucleus was stained by DAPI (blue). Scale bars, 100 μm. *B*: the protein level of p63 and phosphorylated checkpoint kinase 2 (p-CHK2) was examined by Western blotting after *Ash1l* overexpression. Every group was 10 ovaries, repeated at least 3 times. *C*: the protein level of p-CHK2. *n* = 3. *D*: the protein level of p63. *n* = 3. The data were analyzed by *t* test and 2-way ANOVA. Data are shown as means ± SD and considered statistically significant at *P* < 0.05.

Since there was a deficiency in DSB repair and increased apoptosis in fetal oocytes after *Ash1l* was overexpressed, DNA damage checkpoint signaling was presumed to be impaired in these fetal oocytes. Accordingly, phosphorylated ataxia telangiectasia mutated kinase (p-ATM) showed notable increased expression in *Ash1l* overexpression ovaries and was primarily located within the oocyte nucleus (Fig. 7*A*). Consistently, phosphorylated checkpoint kinase 2 (p-CHK2) was also upregulated when *Ash1l* was overexpressed. Intriguingly, as the major downstream effector that is required for culling the oocytes bearing unrepaired DSBs, p63 was increased consistently with p-CHK2 (Fig. 7, *B*–*D*, Supplemental Fig. S2). Together, these results showed that *Ash1l* overexpression causes DNA damage and upregulated p63 and p-CHK2 levels cause these oocytes that have DNA damage to go to apoptosis.

## DISCUSSION

Our results from this study revealed the functional role of ASH1L in controlling early oogenesis and folliculogenesis in fetal ovaries in mice. Through a series of experiments, we clarified that ASH1L expression levels in ovary regulate the integrity of oocyte genomes in meiotic prophase I. Overexpression of *Ash1l* leads to aberrantly high expression of p63 in fetal oocytes. *Ash1l* probably affects the transcription of *p63* in ovary. In addition, the appropriate level of ASH1L is essential for primordial follicle formation and ensures oocyte quality in mice.

ASH1L is widely expressed in various tissues, mainly expressed in the brain, heart, and kidney (26). As a histone methyltransferase, ASH1L regulates many cellular responses and is associated with active transcription (27–29). In the reproductive system, ASH1L has been reported to regulate uterine and paratyphoid development by altering *Hox* gene expression. Moreover, *Ash1l* mutation mouse ovaries have all the stages of folliculogenesis, and the ovaries also produce corpora lutea, consistent with ovulation. The oocytes from mutation mice could be cultured to the blastocyst stage, and there is no difference in fertilization rates (23). In this work, the results showed that ASH1L was expressed in the nucleus of both oocytes and somatic cells in newborn mice ovaries. In our previous work, we found that knockdown of *Ash1l* in 16.5 dpc ovaries resulted in increased oocyte numbers and an enlarged pool of primordial follicles (7). However, the downstream pathway and mechanism are not clear. In this study, we found that overexpression of *Ash1l* reduced the number of primordial follicles and upregulated apoptosis-related proteins. It was further found that after overexpression of *Ash1l* oocyte meiosis was abnormal and oocyte quality guardian protein p63 was significantly increased. These studies suggest that ASH1L may influence oocyte fate by controlling the meiosis process. It also suggests that although both ASH1L and Lysine-specific histone demethylase 1 (LSD1) regulate H3K4me2, their mechanisms are different, which also reflects the complexity of the regulation mechanism of primordial follicle formation.

Oocyte DSBs initiate from leptotene stage in meiotic prophase I. ATM kinase performs an essential role in controlling DSB numbers and shaping DSB distribution. The tolerance of oocytes to DSBs is different at different stages. Postpartum oocytes in mice are more sensitive to DSBs, and when DSB levels are high, programmed death is triggered to remove defective oocytes (30, 31). Unrepaired meiotic DSBs or induced DSBs could cause oocyte elimination and female infertility by triggering the DNA damage response pathway. From our results, the levels of γH2AX were significantly elevated in *Ash1l* overexpression oocytes indicating accumulated DSBs. After CHK2 receives DSBs signals, it transmits DNA damage signals to p63 in oocytes. p63 has been shown to be modulated by CHK2 and casein kinase 1 (CK1) actions, which orchestrate the induction of apoptosis in oocytes. Taken together, our data demonstrate that *Ash1l* overexpression resulted in DSB accumulation and severely impaired oocyte quality and activated the DNA damage response pathway in oocytes, which induces apoptosis (32, 33). In this study, *Ash1l* overexpression resulted in the upregulation of p63 at the mRNA and protein levels, simultaneously leading to oocyte loss in fetal ovaries. The increase of p63 at the mRNA and protein levels may be due to the promotion of *p63* transcription after *Ash1l* overexpression.

ASH1L is an epigenetic factor important for activating gene expression during development. Studies have shown that loss of ASH1L impaired the expression of genes that are critical for brain development (34, 35). Further experiments also demonstrated that ASH1L can epigenetically regulate the expression of essential osteogenic and chondrogenic transcription factors. It exerts this impact by modifying the enrichment of H3K4me3 on their promoter regions (36). In addition, ASH1L can promote the recruitment of lens epithelium-derived growth factor (LEDGF) and other proteins in the chromatin of key target genes of leukemia and is a key regulator of mixed-lineage leukemia (MLL)-dependent transcription and leukemia transformation (37). In our study, we examined genes involved in meiosis-specific programmed DSB formation and recombination, such as *Dmc1*, *Rad51*, *Rec8*, *Spo11*, *Msh4*, and *Stag3*. The results showed that the transcription levels of these genes were significantly increased. A disorder in the expression of these genes is likely to result in meiotic progression delay and meiotic DSB repair deficiency in fetal oocytes in mice. Our results showed that the transcription level of p63 was significantly upregulated. Combining our results with other people’s reports, we hypothesize that the upregulation of these genes was due to the epigenetic regulation by ASH1L.

Upon the disrupted DNA damage checkpoint expression in ovaries following *Ash1l* overexpression, massive oocyte attrition occurred through the apoptosis pathway. Based on our observations from Western blotting and immunofluorescence studies, we detected increased expression of oocyte apoptosis signals such as TUNEL-positive signal and active Caspase3-positive signal in *Ash1l-*OE ovaries, which provided additional evidence for reduction of the number of fetal oocytes through the apoptotic pathway. More importantly, we provide evidence that oocyte loss caused by *Ash1l* overexpression could be rescued by using apoptosis inhibitors.

In conclusion, our results indicate that ASH1L is important for early oogenesis and folliculogenesis in mice. These findings could contribute to opening up a new avenue of research to expand our understanding of physiological and pathological processes in the mammalian ovary.

## SUPPLEMENTAL DATA

Supplemental Tables S1 and S2, Supplemental Material, and Supplemental Figures S1 and S2: https://doi.org/10.6084/m9.figshare.20261358.v4.

## GRANTS

This study was funded by the National Natural Science Foundation of China (32100913 to M. He and 32100686 to T. Zhang), Science and Technology Fund Project of Guizhou Provincial Health Commission (gzwkj2021-527 to M. He, gzwkj2021-299 to T. Zhang), Guizhou Provincial Science and Technology Projects (ZK[2022]346 to M. He), China postdoctoral science foundation (2021M700972 to M. He and 2022M710919 to T. Zhang), Excellent Young Talents Plan of Guizhou Medical University (2022110), Doctoral Startup Foundation of Guizhou Medical University ([2020]038 to T. Zhang, [2020]039 to M. He), the National Natural Science Foundation cultivation project of Guizhou Medical University (20NSP031 to T. Zhang, 20NSP008 to M. He), and Innovation and Entrepreneurship Training Program for College Students in Guizhou Province (202110660058).

## DISCLOSURES

No conflicts of interest, financial or otherwise, are declared by the authors.

## AUTHOR CONTRIBUTIONS

T.Z., C.Z., T.C., and M.H. conceived and designed research; T.Z., T.R., H.L., Y.T., J.Z., J.N., Y.Z., Y.W., and M.H. performed experiments; T.Z., B.J., and M.H. analyzed data; T.Z. interpreted results of experiments; T.Z. and M.H. prepared figures; T.Z. and M.H. drafted manuscript; T.Z., C.Z., T.C., and M.H. edited and revised manuscript; T.Z., T.R., H.L., Y.T., J.Z., J.N., Y.W., T.C., and M.H. approved final version of manuscript.
